# Supplementary figures and images for: Developing, Characterizing, and Modeling CRISPR-Based Point-of-Use Pathogen Diagnostics
Source: ACS Synth Biol. 2024 Dec 13;14(1):129–47. doi: 10.1021/acssynbio.4c00469 (PMC11744932; doi:10.1021/acssynbio.4c00469)

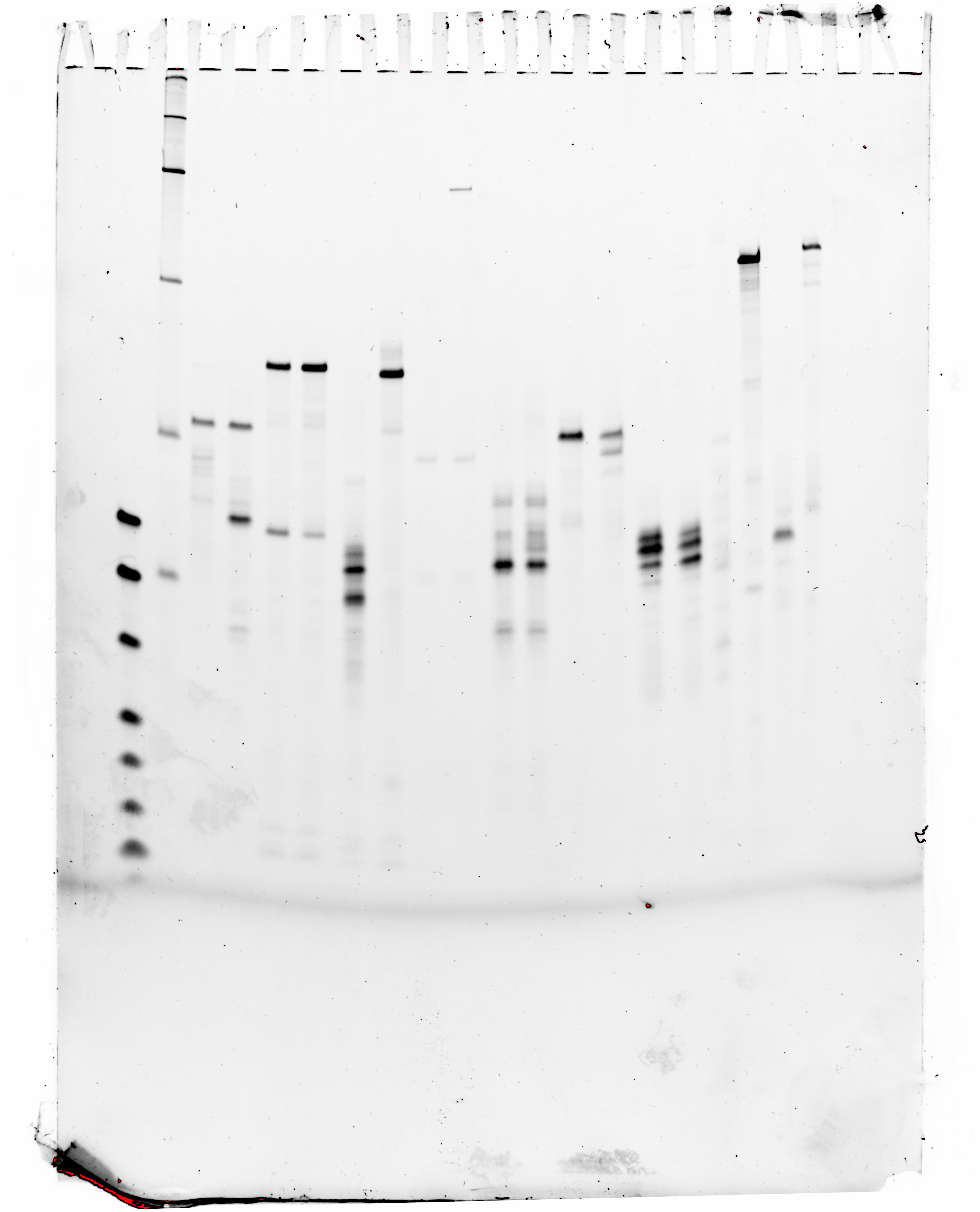

Supplement: Supplementary file 3 — sb4c00469_si_003.zip [file sb4c00469_si_003.zip › Jung_NASBA-Cas13a_Supp_Data_File2_Images/Figure1F/Figure1F_Replicate1.tif]

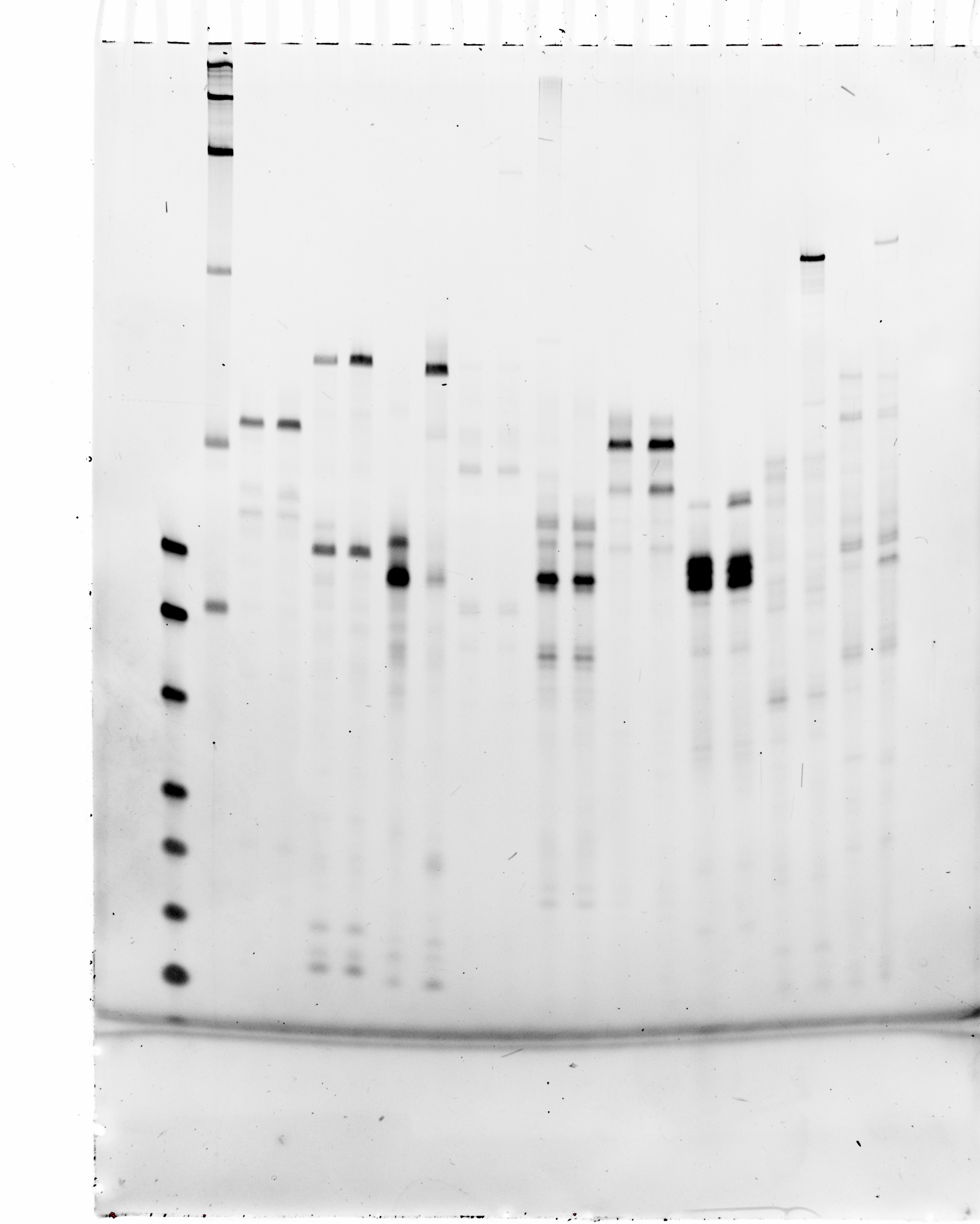

Supplement: Supplementary file 3 — sb4c00469_si_003.zip [file sb4c00469_si_003.zip › Jung_NASBA-Cas13a_Supp_Data_File2_Images/Figure1F/Figure1F_Replicate2.tif]

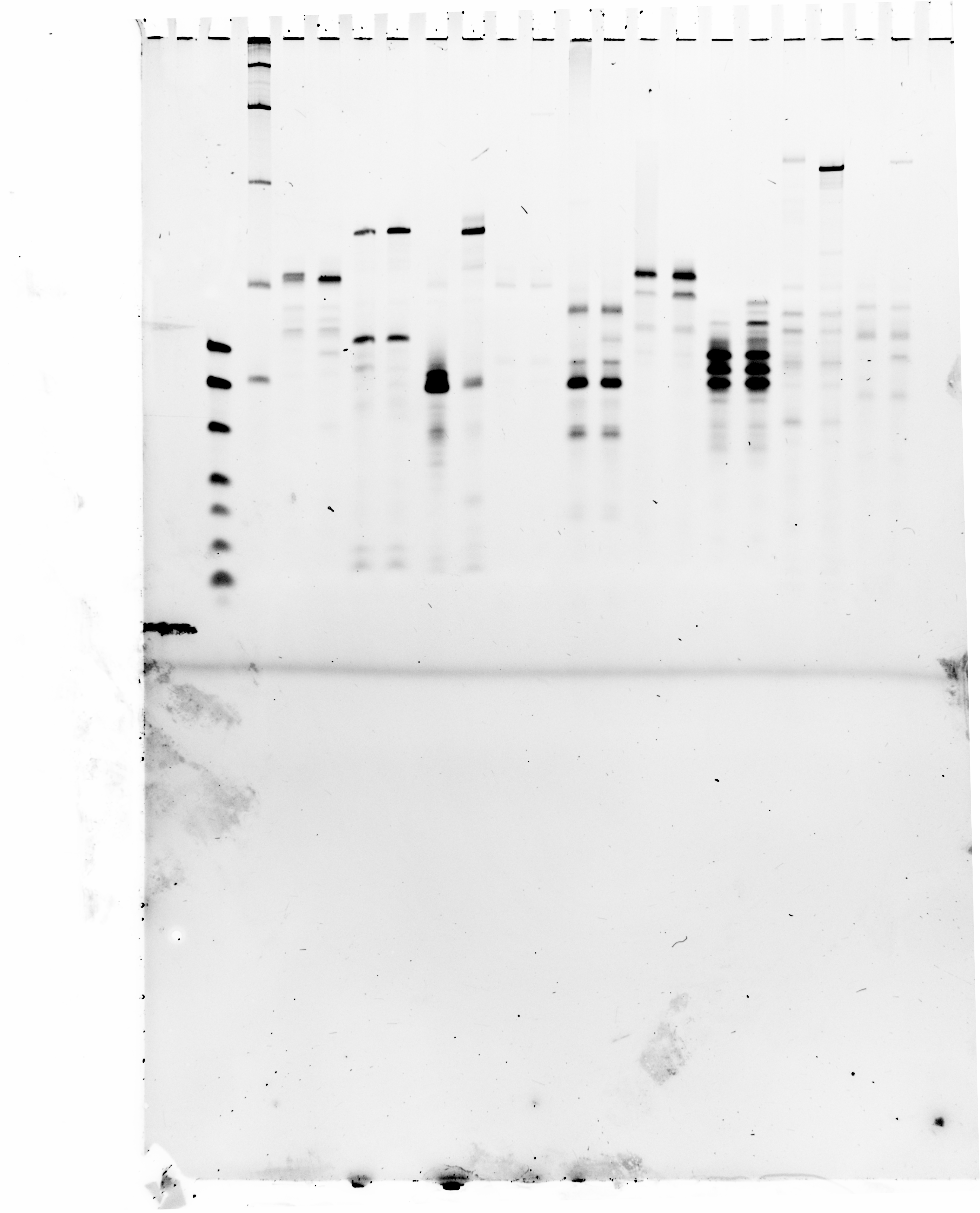

Supplement: Supplementary file 3 — sb4c00469_si_003.zip [file sb4c00469_si_003.zip › Jung_NASBA-Cas13a_Supp_Data_File2_Images/Figure1F/Figure1F_Replicate3.tif]

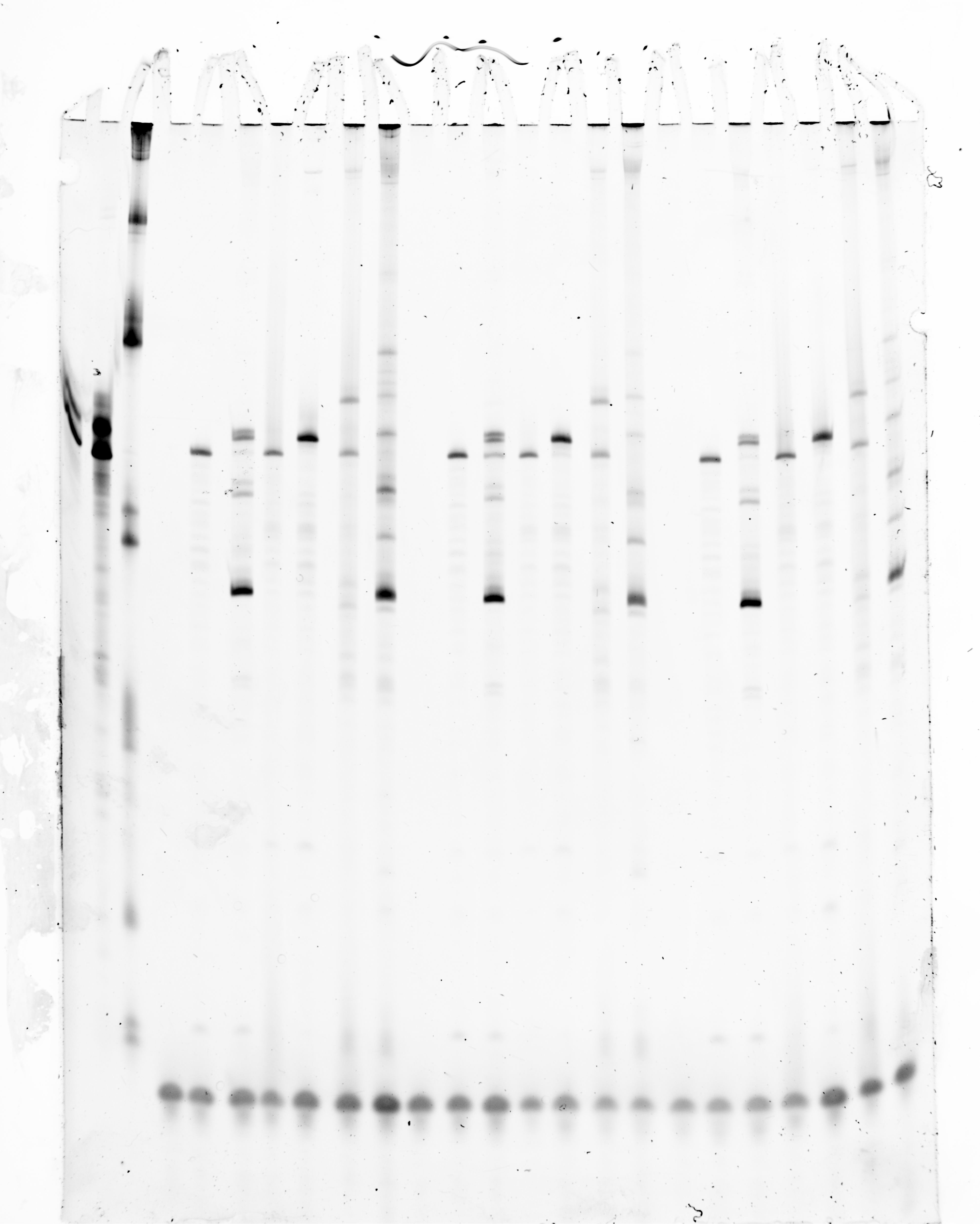

Supplement: Supplementary file 3 — sb4c00469_si_003.zip [file sb4c00469_si_003.zip › Jung_NASBA-Cas13a_Supp_Data_File2_Images/FigureS3A/FigureS3A.tif]

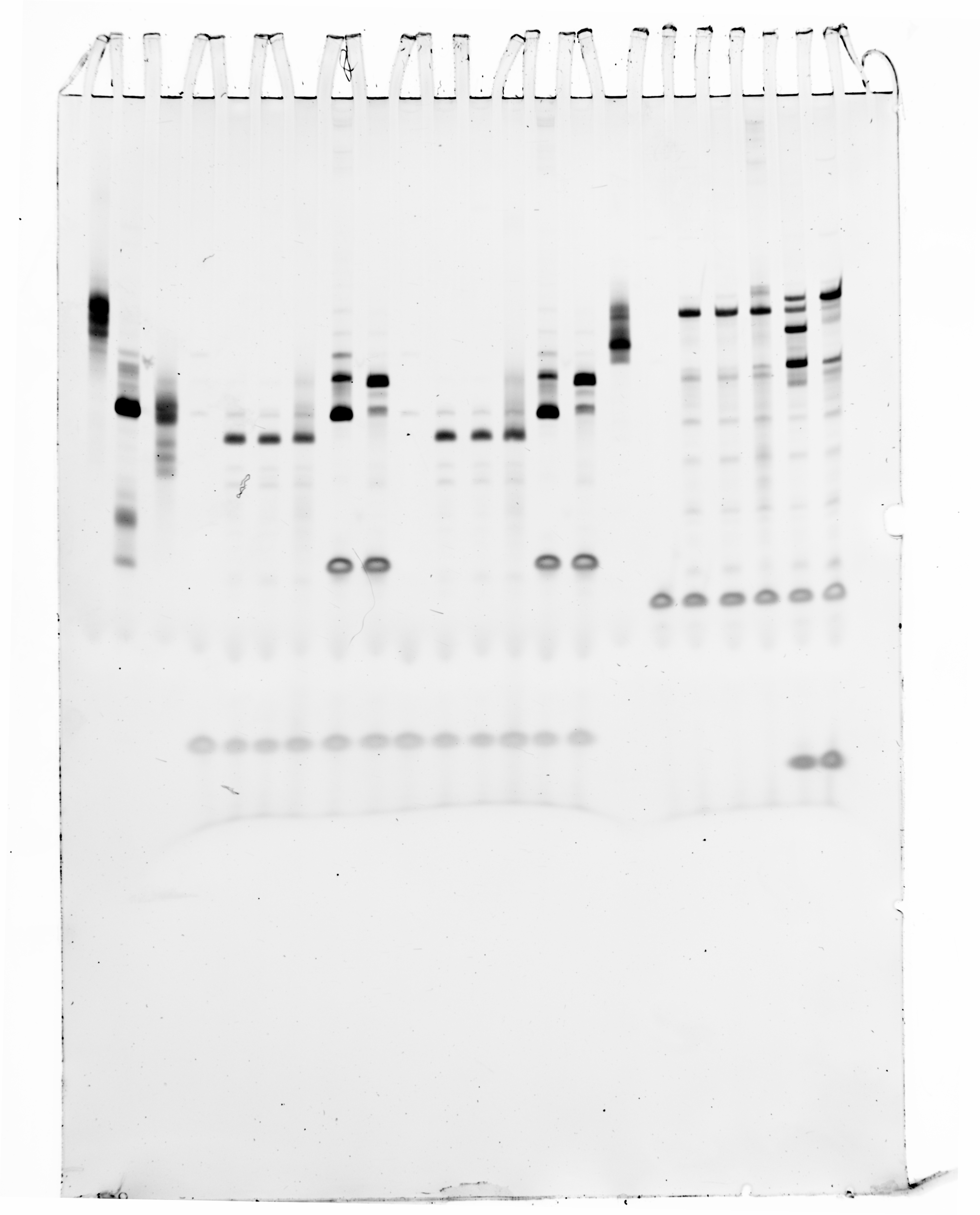

Supplement: Supplementary file 3 — sb4c00469_si_003.zip [file sb4c00469_si_003.zip › Jung_NASBA-Cas13a_Supp_Data_File2_Images/FigureS3B,C/FigureS3B,C.tif]
